# Development and emerging trends in gastrointestinal dysfunction of Parkinson’s disease: a decade-long bibliometric analysis

**DOI:** 10.3389/fnagi.2025.1712302

**Published:** 2025-11-28

**Authors:** Yujia Sun, Haoyu Yang, Junfeng Zhang, Shan Cong, Lin Wang, Tao Yu

**Affiliations:** 1 The First Teaching Hospital of Tianjin University of Traditional Chinese Medicine, Tianjin, China; 2 National Clinical Research Center for Acupuncture and Moxibustion, Tianjin, China; 3 Tianjin University of Traditional Chinese Medicine, Tianjin, China; 4 Tianjin Xiqing District Traditional Chinese Medicine Hospital, Tianjin, China; 5 Tianjin Academy of Traditional Chinese Medicine Affiliated Hospital, Tianjin, China

**Keywords:** Parkinson’s disease, gastrointestinal symptoms, bibliometrics, brain-gut-microbiome axis, global research trends

## Abstract

Gastrointestinal (GI) dysfunction represents a prevalent non-motor symptom of Parkinson’s disease (PD) that not only contributes significantly to disease progression but also substantially compromises patients’ quality of life. Over the past decade, research in this domain has expanded considerably. To systematically delineate the knowledge framework and evolving trends, we performed a bibliometric analysis of publications on GI and PD from 2015 to 2025. A total of 924 articles were retrieved from the Web of Science Core Collection (WoSCC). Co-occurrence, clustering, and collaboration network analyses were performed using VOSviewer, CiteSpace, and the R package Bibliometrix. For findings validation, the PubMed database was incorporated as an independent external validation dataset, providing complementary verification of keyword analyses derived from WoSCC. Our analysis revealed a steady annual increase in publication output. China and the United States emerged as the most prolific contributors globally, with the latter attaining the highest total citation count. At the institutional level, Capital Medical University led in publication output, whereas the University of Helsinki ranked highest in both total and average citations. Among journals, *Parkinsonism & Related Disorders* published the most papers on this topic, while *Movement Disorders* received the most citations. Keyword cluster analyses identified three primary research frontiers: (1) pathogenesis, focusing on α-synuclein (α-syn), the brain-gut-microbiome axis, and the enteric nervous system; (2) clinical manifestations, especially dysphagia and constipation; and (3) therapeutic interventions, particularly fecal microbiota transplantation and probiotics. By integrating established knowledge and highlighting emerging trends, this review aims to inform and guide future research and clinical practice in the field of gastrointestinal dysfunction in PD.

## 1 Introduction

Parkinson’s disease (PD) is the second most prevalent neurodegenerative disorder worldwide, with its associated burden rising in parallel with global population aging (Dorsey et al., 2018). The 2021 Global Burden of Disease (GBD) study projects that approximately 25.2 million individuals will be affected by PD by 2050 (Su et al., 2025). The classic pathological hallmarks of PD include degeneration of dopaminergic neurons in the substantia nigra pars compacta, a brain region critical for motor control, and abnormal accumulation of α-synuclein (α-syn) protein within the central nervous system, which comprises the brain and spinal cord (Morris et al., 2024). Notably, recent studies have detected pathological α-syn in peripheral tissues, especially within the enteric nervous system, the neuronal network of the gastrointestinal tract. This finding challenges the conventional view of PD pathogenesis (Yang and Zhang, 2024).

The clinical manifestations of PD encompass both motor symptoms—such as resting tremor, bradykinesia, rigidity, and postural instability—and a spectrum of non-motor symptoms (Mammen et al., 2025). Gastrointestinal (GI) dysfunction is a common condition that affects the entire GIT. Manifestations such as sialorrhea, dysphagia, gastroparesis, and constipation can significantly impact patients’ quality of life. GI disturbances may alter levodopa pharmacokinetics and raise the risk of complications, such as malnutrition and aspiration pneumonia (Warnecke et al., 2022; Leta et al., 2023). Approximately 67.5% of PD patients experience at least one GI symptom prior to the onset of overt motor signs, which highlights GI dysfunction as a potential prodromal marker (Durcan et al., 2019). The “gut-first” hypothesis, proposed by Braak et al., posits PD may originate in the gut and spread to the brain via the vagus nerve (Braak et al., 2003a). This concept contrasts with the traditional view, which holds the central nervous system as the initial site of pathology, followed by peripheral spread (Horsager et al., 2022). These competing ideas have significantly advanced research into the pathophysiology of PD (Horsager and Borghammer, 2024; Nagaraj et al., 2025).

Advances in high-throughput genomic sequencing have clarified the pivotal role of the microbiota–gut–brain axis in PD (Chen et al., 2025). Accumulating evidence suggests that gut microbiota and their metabolites influence PD initiation and progression via mechanisms such as immune-inflammatory regulation, maintenance of intestinal barrier integrity, and neurotransmitter systems (Wang et al., 2021; Liu et al., 2025). However, this field faces several challenges. High inter-individual variability in gut microbiota, together with confounders such as geography and diet, complicates the identification of consistent PD-specific microbial signatures (Ma, 2020). Furthermore, many clinical studies are limited by small sample sizes, short durations, and lack of standardized methodologies. Collectively, these factors hamper the reliability and generalizability of their findings.

Previous bibliometric analyses have provided a broad overview of non-motor symptoms in PD. Nevertheless, a focused investigation specifically addressing the rapidly expanding research on GI symptoms remains lacking (Li et al., 2024). To address this gap, this study represents the first bibliometric analysis systematically evaluating research output, collaboration patterns, and emerging trends in PD-related GI dysfunction from 2015 to 2025. We aim to map the field’s evolution and provide an evidence-based roadmap for future mechanistic studies and clinical interventions.

## 2 Materials and methods

### 2.1 Data retrieval and collection

A dual-database search strategy was employed to leverage the complementary strengths of different bibliographic sources and to validate the robustness of the results. The Web of Science Core Collection (WoSCC) was selected as the primary data source, while PubMed served as an independent dataset for external validation. WoSCC is internationally recognized for its extensive coverage of high-impact journals and its unique citation indexing capabilities. Conversely, PubMed, a leading resource in biomedical literature, excels at identifying emerging research fronts through its structured Medical Subject Headings (MeSH) vocabulary and frequent updates (Falagas et al., 2008). The search covered publications from January 1, 2015, to August 6, 2025. Only records classified as “Article” or “Review” and published in English were included.

#### 2.1.1 WoSCC database search

Data were retrieved from the WoSCC, specifically the Science Citation Index Expanded (SCI-EXPANDED) and the Social Sciences Citation Index (SSCI). The search strategy was defined as follows: TS = ((Parkinson OR “Parkinson’s Disease”) AND (“gastrointestinal*” OR “gastropares*” OR “gastric stasis” OR “delayed gastric emptying” OR “constipat*” OR “Dyschezia” OR “sialorr*” OR “hypersaliv*” OR “”drool*” OR “deglutition disorder*” OR “swallow* disorder*” OR “dysphag*”)). This initial search returned 936 records. Full bibliographic metadata, including cited references, was downloaded in plain text for subsequent analysis.

#### 2.1.2 PubMed database search

The search strategy for PubMed was designed to be analogous to the WoSCC approach and incorporated the precision of MeSH terms. The detailed search strategy was as follows. (“Parkinson Disease” [Mesh]) OR (Parkinson [Title/Abstract] OR “Parkinson’s Disease” [Title/Abstract]) AND ((“Gastrointestinal Diseases” [Mesh]) OR (“Gastroparesis” [Mesh]) OR (“Gastric Stasis” [Title/Abstract] OR “delayed gastric emptying” [Title/Abstract]) OR (“Constipation” [Mesh] OR constipat* [Title/Abstract]) OR (“Dyschezia” [Title/Abstract]) OR (“Sialorrhea” [Mesh] OR sialorr* [Title/Abstract] OR hypersaliv* [Title/Abstract] OR drool* [Title/Abstract]) OR (“Deglutition Disorders” [Mesh] OR “deglutition disorder*” [Title/Abstract] OR “swallow* disorder*” [Title/Abstract] OR dysphag* [Title/Abstract])). This search yielded 403 relevant publications, and all complete data records were exported in PubMed format for storage.

### 2.2 Data standardization

**FIGURE 1:**
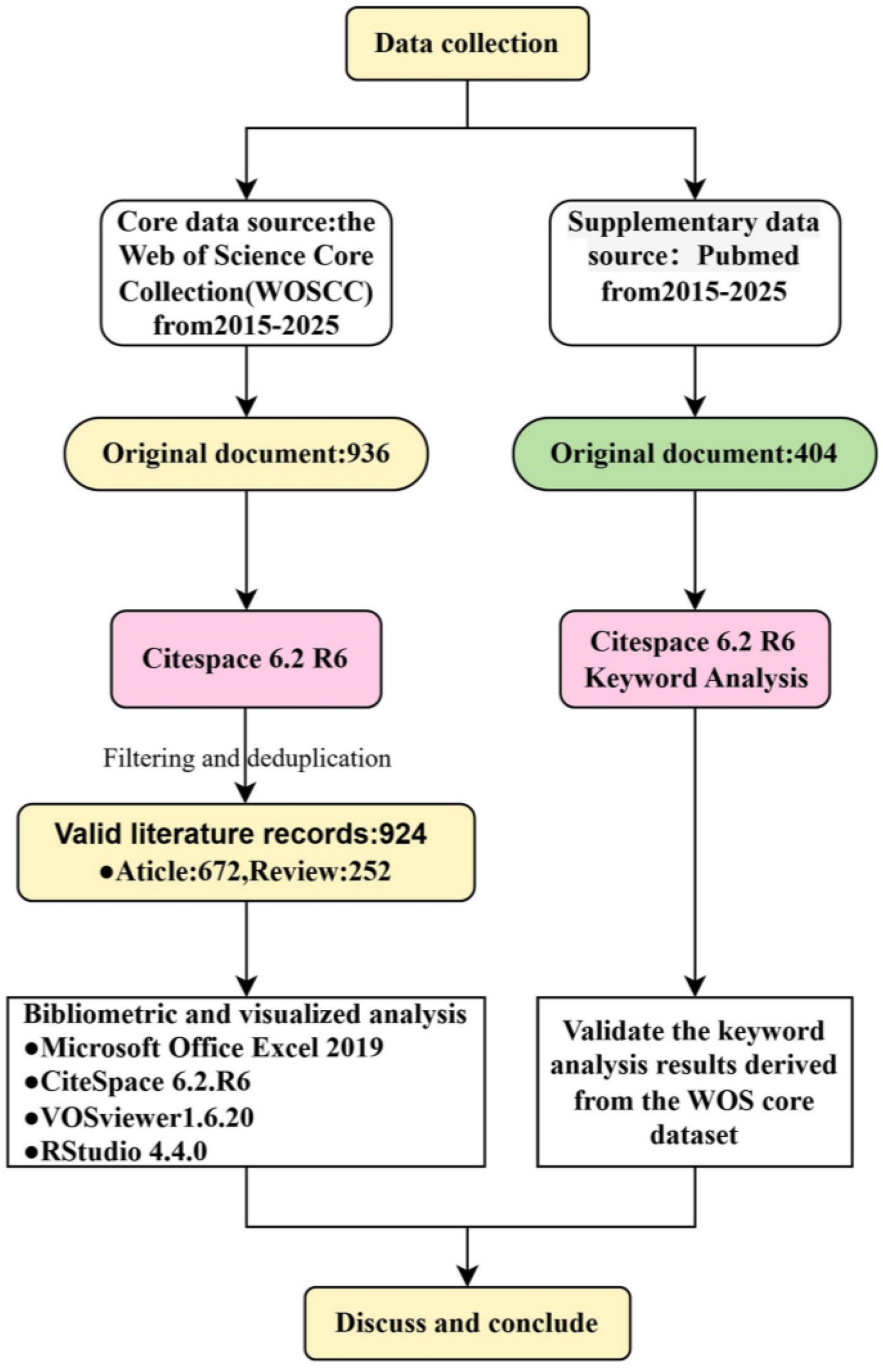
Flow chart of the literature search and screening.

A standardized data-cleaning procedure was applied independently to both the WoSCC and PubMed datasets to ensure data quality and consistency. This process involved deduplication and the normalization of variant terms for keywords, author affiliations, and countries. Following data cleaning, the final WoSCC dataset comprised 924 articles, while the PubMed dataset contained 403 articles, which were subsequently designated as an external validation set. A comparative analysis of high-frequency keyword clusters between the two datasets was conducted to evaluate the congruence of central research themes. This approach served to evaluate the stability of the research fronts and trends identified from the primary WoSCC dataset. The overall data flow and inclusion criteria are depicted in Figure 1.

### 2.3 Data analysis

The WoSCC dataset served as the primary source for this bibliometric analysis. The analytical tools applied and their specific functions are summarized in Table 1

**TABLE 1:** Tools and function.

Tools	Analysis function	Key parameter settings
Excel (2019)	Annual publication trend chart	–
CiteSpace (version 6.2.R6)	Burst detection; Dual-graph superposition of journals; Document co-citation analysis Keyword clustering analysis	Time slicing: 2015–2025 (slice length = 1 year); Node selection criteria: g-index (*k* = 8)
VOSviewer (version 1.6.20)	Countries/institutional collaboration network; Keyword co-occurrence network	Minimum publication threshold for countries/institutions; Minimum frequency threshold for keywords: ≥ 10
Package “Bibliometrix” (version 4.3.0, RStudio 4.40)	Trend topics	–
Scimago Graphica	Global geographic visualization of publications	–
Journal Citation Reports	Obtain 2024 journal impact factor (IF)	–

To complement and verify the consistency of the keyword hotspots identified from WoSCC, an independent dataset from PubMed was incorporated. This supplementary dataset was processed using VOSviewer (version 1.6.20) to construct a comparable keyword co-occurrence network under identical analytical parameters (minimum occurrence threshold = 10). Subsequently, keyword cluster analysis was performed utilizing CiteSpace (version 6.2.R6). The robustness of the findings was objectively evaluated through a comparative examination of keyword hotspots and cluster themes derived from both databases.

## 3 Results

### 3.1 Annual trends in publications and citations

**FIGURE 2:**
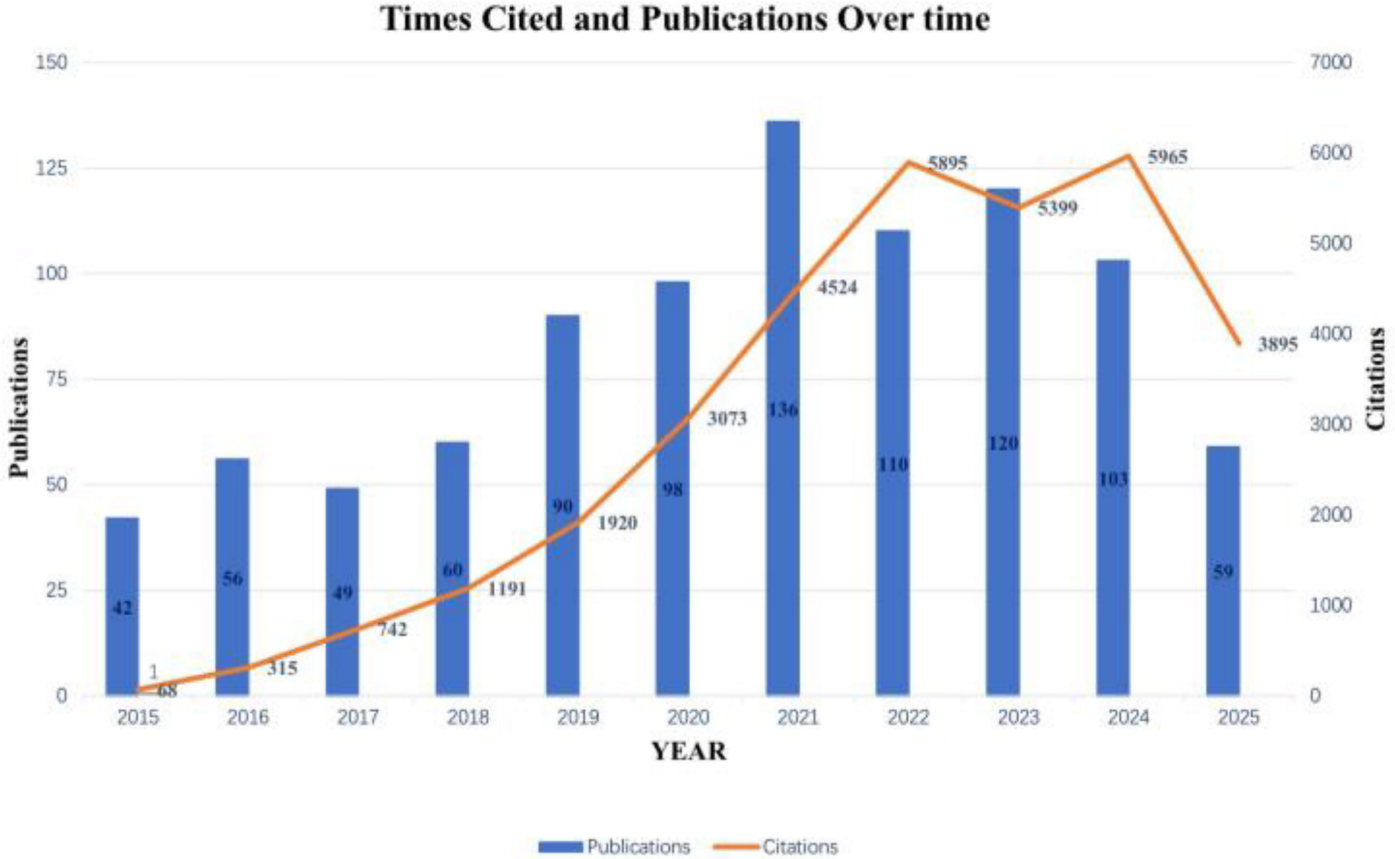
Annual publication trends in Parkinson’s disease-related gastrointestinal dysfunction (2015–2025).

Analysis of publication outputs from 2015 to 2025 revealed a substantial increase in research activity focusing on GI dysfunction in PD (Figure 2). The annual publication count rose from 42 in 2015 to a peak of 136 in 2021, representing a 2.2-fold growth over this period. Although a slight decline occurred between 2022 and 2024, the annual output stabilized at an average of 111 publications—significantly elevated compared to the baseline level of 49 publications per year recorded during 2015–2017. Notably, publications from 2025 alone had garnered 3,895 citations by the time of data retrieval, underscoring the sustained expansion and growing impact of research in this field.

### 3.2 Contributions by country and institution

**FIGURE 3:**
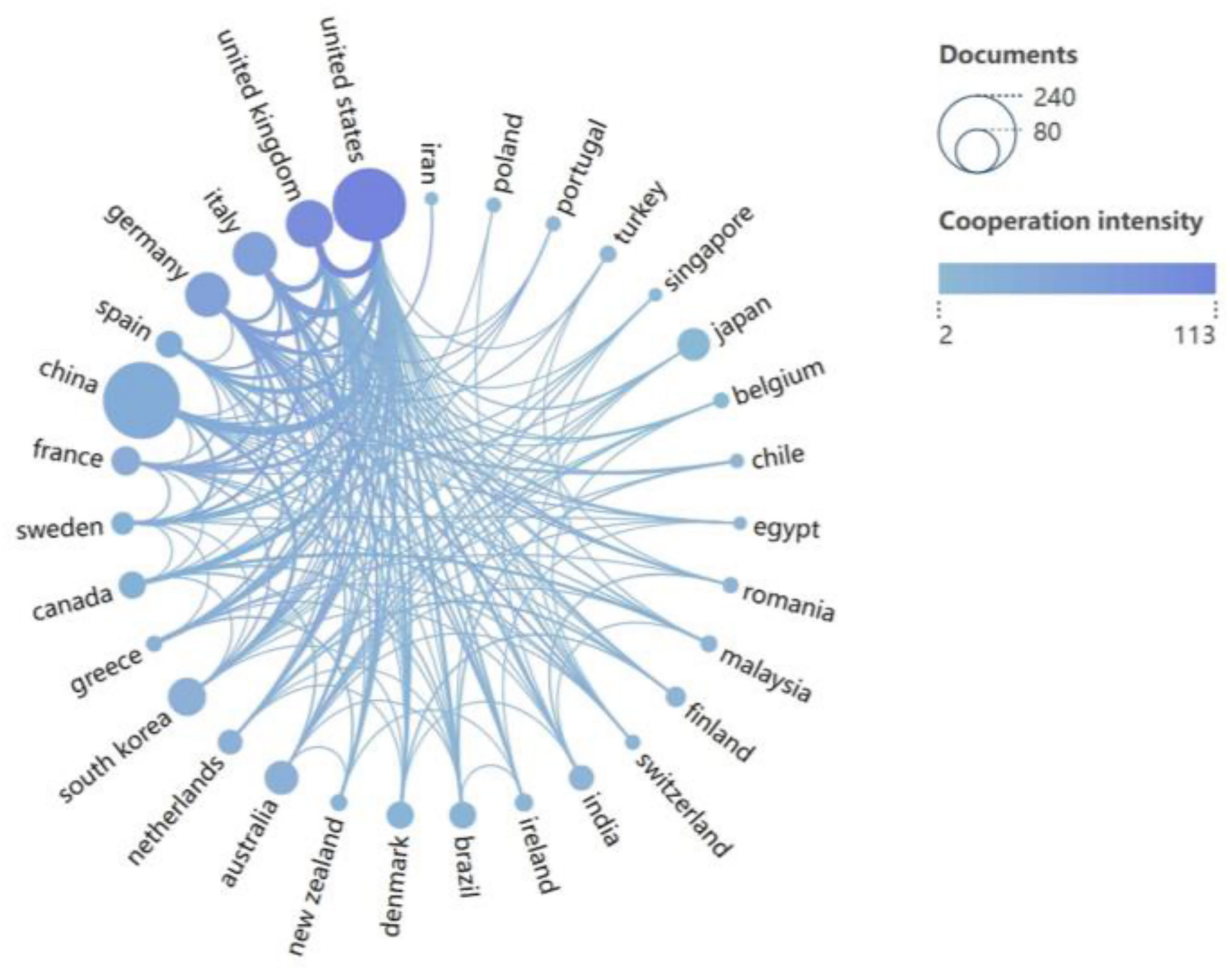
Collaboration network of 31 countries (publications > 5). Node size represents publication count; line color intensity indicates collaboration strength.

A total of 62 countries/regions contributed to the research field of PD-associated GI dysfunction, with the global distribution of productivity detailed in Table 2. In terms of publication volume, China (*n* = 221) and the United States (*n* = 201) emerged as the dominant contributors, each producing more than double the output of the third-ranked country, the United Kingdom (*n* = 84). Regarding research impact, the United States led in total citation count (8,722), followed by China (6,359) and Germany (4,723). More indicative of research influence, however, was the average number of citations per article. Finland ranked first globally (145.8), highlighting its exceptionally high research impact. In contrast, China’s average citation rate (28.8) was substantially lower than the mean for the top 10 countries (Supplementary Appendix 1) suggesting room for improvement in research quality and international visibility. The international collaboration network (Figure 3) identified the United States (total link strength = 115) and the United Kingdom (total link strength = 98) as central hubs, demonstrating extensive and robust collaborative ties with numerous other countries.

**TABLE 2:** Top 10 most productive countries in PD-related GI dysfunction.

Country	Articles	Citations	Average article citations	Total link strength
China	221	6,359	28.8	34
United States	201	8,722	43.4	115
United Kingdom	84	2,940	35.0	98
Germany	75	4,723	63.0	57
Italy	73	2,556	35.0	60
South Korea	54	1,242	23.0	21
Australia	43	1,199	27.9	21
Japan	40	1,439	36.0	7
France	32	1,821	57.0	32
Canada	28	1,999	71.4	34

**FIGURE 4:**
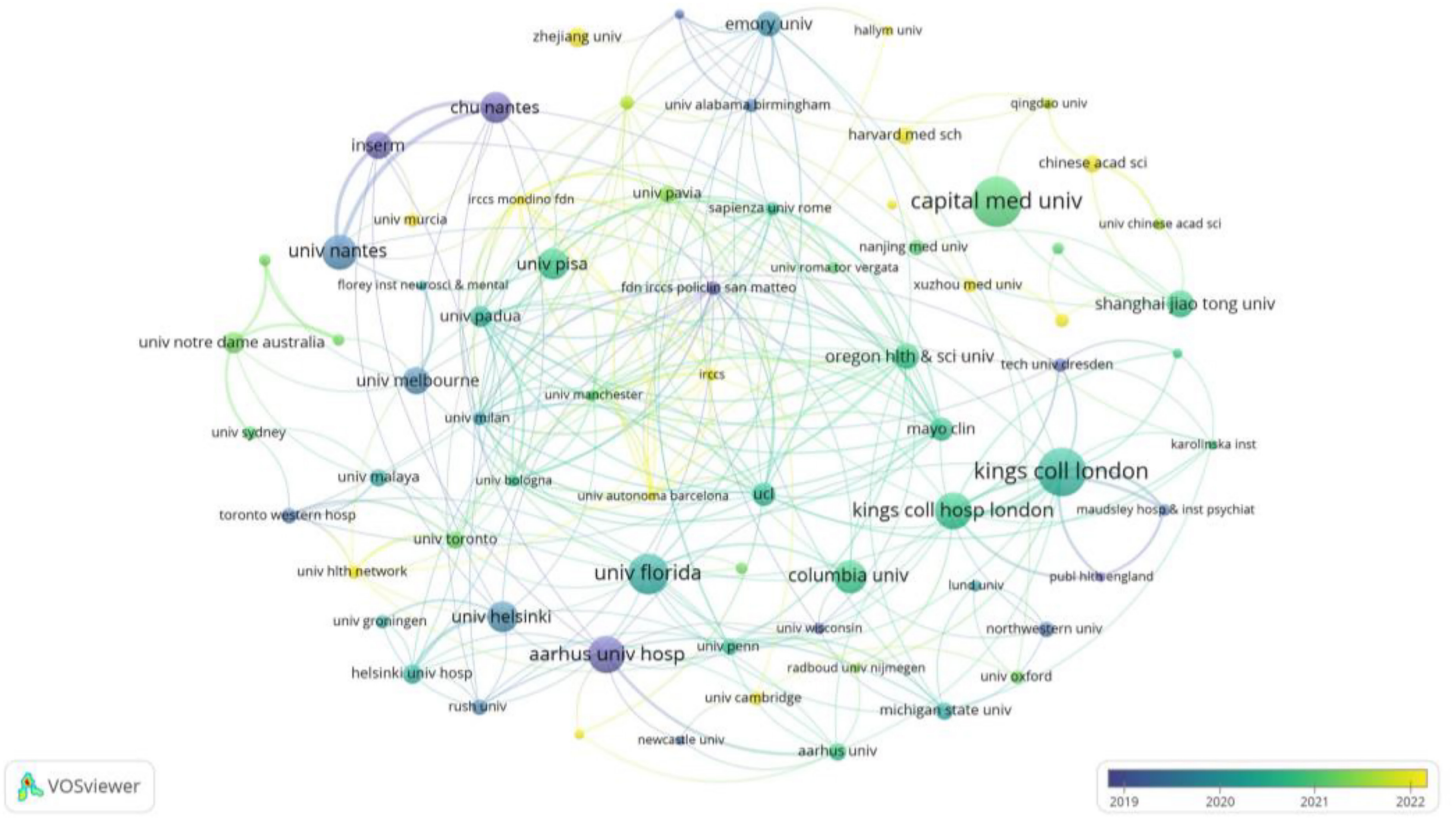
Network diagram illustrating collaborations among 90 institutions (publications > 5). Node size reflects institutional publication volume, while the quantity of links represents the scope of their collaborative partnerships. The strength of inter-institutional collaboration (total connection strength) is shown in Table 3.

At the institutional level, a total of 1,406 institutions worldwide contributed to publications in this field. As presented in Table 3, the top 15 most productive institutions collectively accounted for 368 articles, representing 39.8% of the total output. Among them, University College London (26 papers) was the most productive institution, followed closely by King’s College London (25 papers). Assessment of research impact revealed the University of Helsinki as the most influential institution, achieving the highest metrics in both total citations (2,334) and average citations per paper (145.9). Figure 4 presents the collaborative network among the top 90 institutions (≥5 publications), with the intensity of inter-institutional cooperation quantified by total link strength. Notably, King’s College London (total link strength = 45) and King’s College Hospital London (total link strength = 43) functioned as central nodes in this network, demonstrating their pivotal role in fostering inter-institutional collaborations.

**TABLE 3:** Top 15 publishing institutions in PD-related GI dysfunction.

Affiliation	Articles	Citations	Average article citations	Total link strength
Capital Medical University	26	385	14.8	2
King’s College London	25	877	35.1	45
University of Florida	21	595	28.3	13
Aarhus University Hospital	19	1453	76.5	16
King’s College Hospital London	19	601	31.6	43
University of Nantes	18	925	51.4	30
Columbia University	17	659	38.8	9
Centre Hospitalier Universitaire de Nantes	16	969	60.6	32
University of Helsinki	16	2,334	145.9	20
University of Pisa	16	370	23.1	11
Institut National de la Santé et de la Recherche Médicale	14	863	61.6	28
Shanghai Jiao Tong University	14	1,658	118.4	9
University of Melbourne	14	306	21.9	10
Emory University	13	1,490	114.6	20
Oregon Health & Science University	13	870	67.0	32

### 3.3 Journal contributions and impact analysis

Between 2015 and 2025, a total of 309 academic journals published research on GI dysfunction in PD. Among these, 40 journals contributed five or more articles. Table 4 presents the top 10 journals based on publication output. In terms of publication volume, *Parkinsonism & Related Disorders* (48 articles) and *Journal of Parkinson’s Disease* (46 articles) stood out as the leading platforms. Regarding citation impact, *Movement Disorders* was the clear leader, accumulating 3,899 citations with an average of 150 per article, highlighting its significant influence in the field.

**TABLE 4:** Top 10 journals with the most published articles.

Journals	Documents	Citations	IF
Parkinsonism and Related Disorders	48	2,754	3.4
Journal of Parkinson’s Disease	46	1,478	5
Dysphagia	37	918	3
NPJ Parkinson’s Disease	30	1,217	8.2
Frontiers in Neurology	29	619	2.8
Neurogastroenterology and Motility	27	495	2.9
Movement Disorders	26	3,899	7.6
Neurological Sciences	19	175	2.4
Frontiers in Aging Neuroscience	18	652	4.5
International Journal of Molecular Science	15	538	4.9

Journal co-citation analysis (Supplementary Appendix 2), a method instrumental in delineating foundational knowledge within a field, further reinforced the leading status of *Movement Disorders* (4,804 co-citations) and *Parkinsonism & Related Disorders* (2,515 co-citations), underscoring their role as the core intellectual framework underpinning this research domain. Importantly, the inclusion of high-impact general neurology journals—*Neurology* (1,612), *Lancet Neurology* (728), and *Annals of Neurology* (648)—among the most frequently co-cited sources underscores the broader recognition of GI dysfunction in PD as a scientifically significant and rapidly advancing field.

**FIGURE 5:**
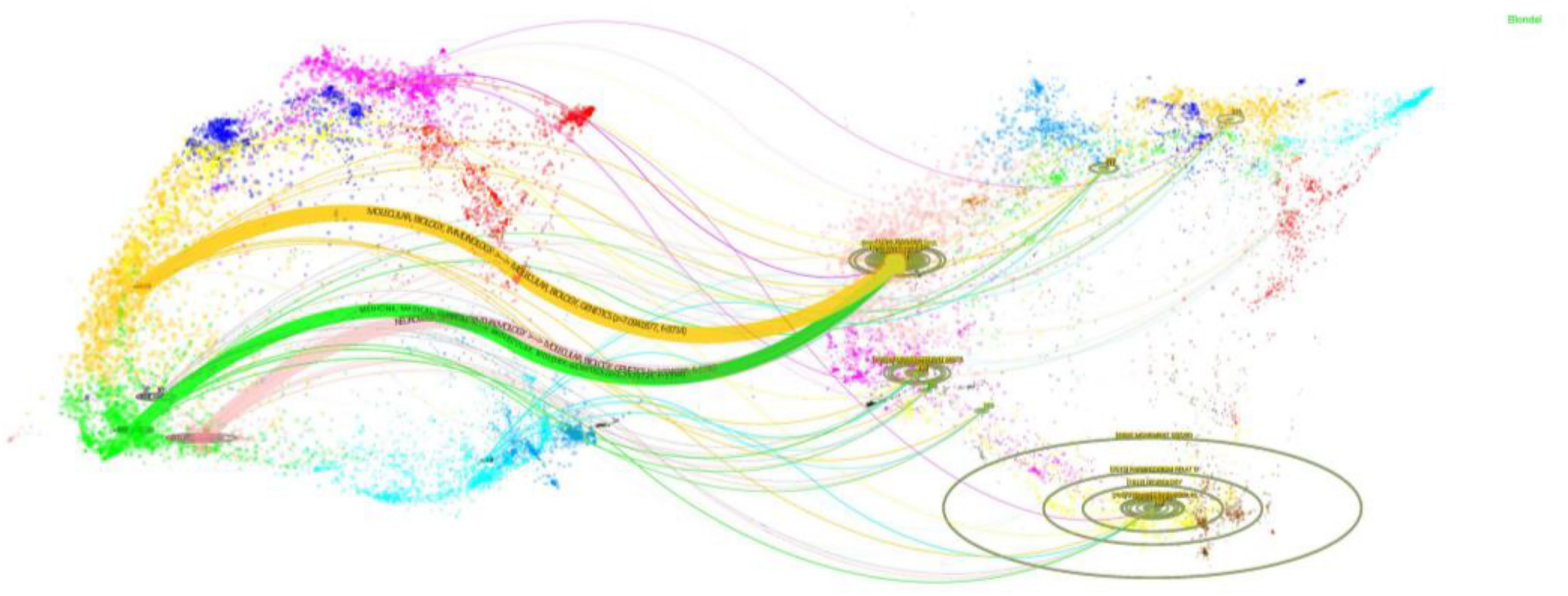
The dual-map overlay illustrates PD-related GI dysfunction research. The left cluster represents citing journals, while the right cluster shows cited journals. Curves connecting the two clusters represent citation pathways. The vertical axis of each ellipse corresponds to the number of articles published in each journal, while the horizontal axis reflects the diversity of contributing authors.

The dual-map overlay analysis (Figure 5) reveals a dynamic knowledge flow in the research of GI dysfunction in PD. As reflected by citing journals, current studies in this field primarily emerge from three domains: Molecular Biology/Genetics, Health/Nursing/Pharmacy, and Medicine/Medical/Clinical. Notably, these research efforts are anchored in a core knowledge base derived from the broader fields of Molecular/Biology/Immunology. The resulting research outputs, in turn, make significant contributions to three key application areas: the foundational Molecular/Biology/Immunology cluster (yellow), the Medicine/Medical/Clinical cluster (green), and the Neurology/Sports/Ophthalmology cluster (pink). This pattern demonstrates a multidisciplinary research paradigm that effectively translates molecular-level discoveries into clinical practice.

### 3.4 High-impact reference analysis

**FIGURE 6:**
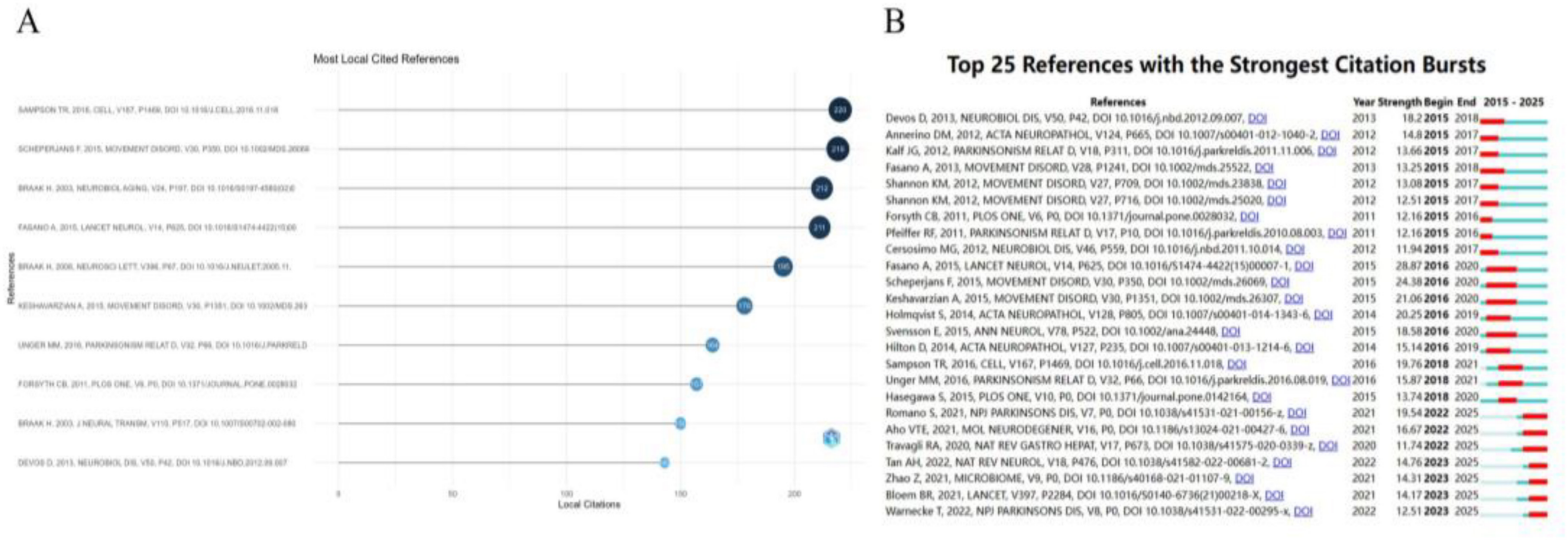
Analysis of co-cited references in PD-related GI dysfunction research. **(A)** Evolution of co-citation trends (2015–2025). **(B)** Top 25 references with strongest citation bursts.

To map the intellectual foundations and evolutionary trajectory of the field, we conducted a co-citation analysis on the 26,296 references extracted from the included studies. Seventeen references accumulated more than 100 citations. Figure 6A lists the top 10 most-cited works (143–220 citations), which collectively constitute the formative literature that has shaped the field. Notably, the collective body of work by Heiko Braak and his team, which amassed 557 citations, stands out; their pioneering theories on PD pathological staging and the gut-origin hypothesis remain a cornerstone of contemporary research in this domain (Braak et al., 2003a; Braak et al., 2003b; Braak et al., 2006). Furthermore, two highly influential studies—by Sampson et al. (2016) (220 citations) and Scheperjans et al. (2015) (219 citations)—have spurred a wave of investigation into the microbiome-gut-brain axis as a critical pathway for understanding PD progression and potential interventions.

A citation burst analysis was performed to identify publications that attracted significant attention over specific periods (Figure 6B). The review by Fasano et al. (2015), published in *The Lancet Neurology* (burst strength = 28.87), was the most prominent. It offered a systematic overview of the pathogenesis, clinical spectrum, and management of PD-related GI dysfunction, establishing it as an essential framework for ongoing research in the field.

### 3.5 Keyword analysis and topic evolution

**FIGURE 7:**
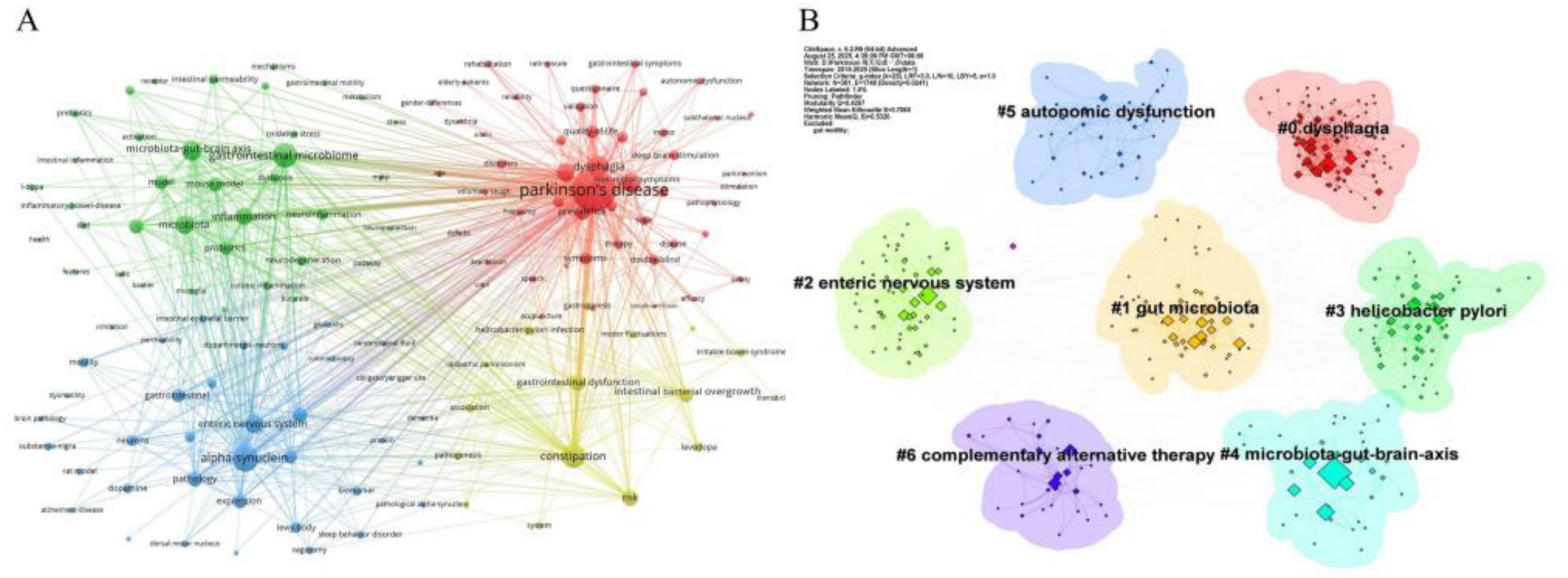
Analysis was performed based on data retrieved from the WOSCC. **(A)** Visualization of the keyword co-occurrence network. The size of each node corresponds to the frequency of the keyword’s appearance. **(B)** Keyword clustering map, which identified seven distinct thematic clusters.

Keyword co-occurrence analysis, a method quantifying the co-occurrence frequency of multiple keywords within the same publications, was employed to reveal the conceptual structure of the research field. As shown in Figure 7A, “Parkinson’s disease” (frequency = 667; total link strength = 3,724) serves as the central node of the network. Other high-frequency keywords include “dysphagia” (226), “constipation” (195), alongside key pathological mechanisms such as “alpha-synuclein” (208), “gastrointestinal microbiome” (191), “microbiota-gut-brain axis” (115), “enteric nervous system” (102), and “inflammation” (100).

Keyword clustering was performed to group terms based on their association strength, thereby identifying central research topics and trends. Employing the log-likelihood ratio (LLR) algorithm in CiteSpace, seven distinct clusters were identified (Figure 7B). The clustering structure exhibits high reliability, with a modularity Q value of 0.4267 and a weighted mean silhouette S value of 0.7085. These clusters can be synthesized into three primary research directions: the largest cluster group (#1, #2, #3, #4) pertains to pathophysiological mechanisms; clusters #0 and #5 relate to clinical manifestations and comorbidities; and cluster #6 focuses on intervention and management strategies.

**FIGURE 8:**
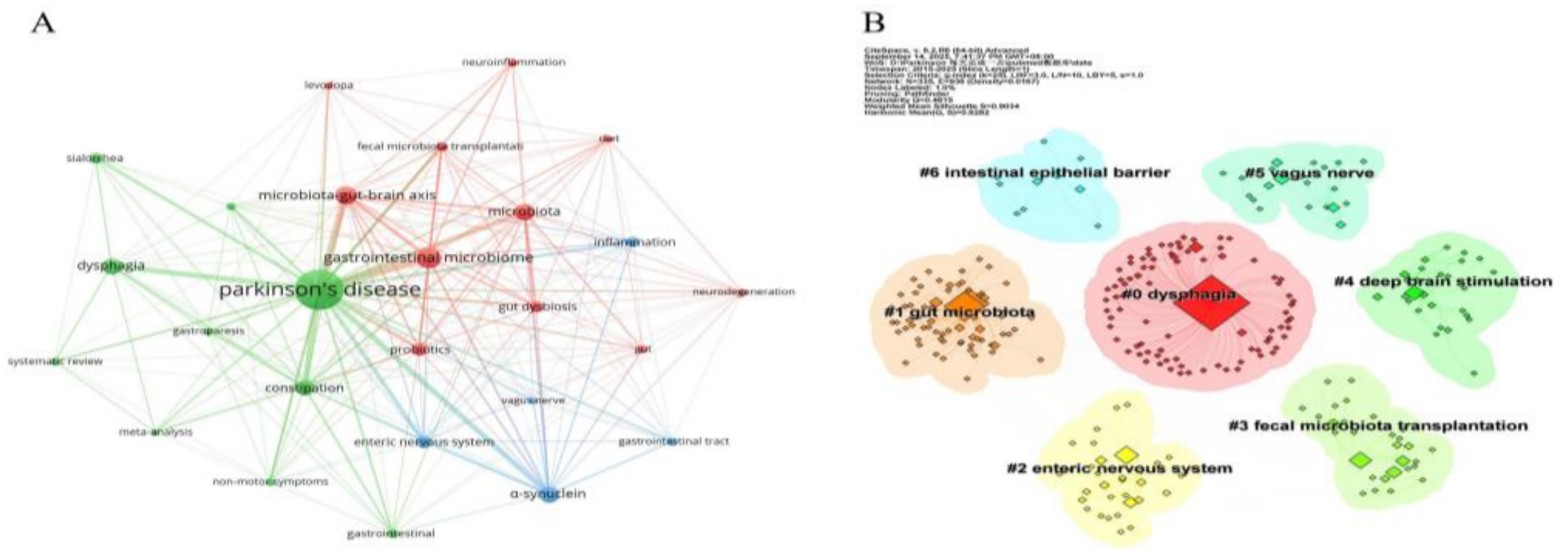
Keyword analysis in the field of PD-associated gastrointestinal dysfunction. **(A)** The co-occurrence network visualization depicts the relationships between keywords, where the size of each node corresponds to the frequency of the keyword’s occurrence. **(B)** The keyword clustering diagram identifies seven distinct thematic clusters.

To validate the reliability of our findings, we conducted a parallel keyword analysis using the PubMed database. The keyword co-occurrence network generated from this dataset (Figure 8A) demonstrated remarkable consistency with the results obtained from the Web of Science (WoS) database. Cluster analysis, performed using the same algorithm, produced seven distinct clusters (Figure 8B) with excellent statistical quality (Modularity Q = 0.4815; Mean Silhouette S = 0.9034). A comprehensive cross-database comparison of the clustering results revealed a high degree of consistency in their core frameworks. Three core clusters identified in the PubMed analysis—“#0 dysphagia,” “#1 gut microbiota,” and “#2 enteric nervous system”—exactly matched the results from WoS. Furthermore, PubMed’s clustering results provided complementary insights to WoS. At the mechanistic level, PubMed’s unique clusters, namely “#5 vagus nerve” and “#6 intestinal epithelial barrier,” offered more precise anatomical and physiological support for gut-brain axis theory. Specifically, the “#5 vagus nerve” cluster directly corroborated its role as a high-speed pathway for the transmission of pathological α-syn between the gut and brain (Han et al., 2025). In contrast, the “#6 intestinal epithelial barrier” cluster emphasized that alterations in intestinal barrier function not only activate systemic inflammatory responses but also exacerbate PD-related GI symptoms (Umamahesan et al., 2025). Regarding intervention strategies, PubMed clusters explicitly identified “#3 fecal microbiota transplantation” and “#4 deep brain stimulation”—two strategies that concretized the relatively broad “#6 complementary alternative therapy” concept identified in WoS, thereby further expanding the scope of alternative therapeutic approaches.

**FIGURE 9:**
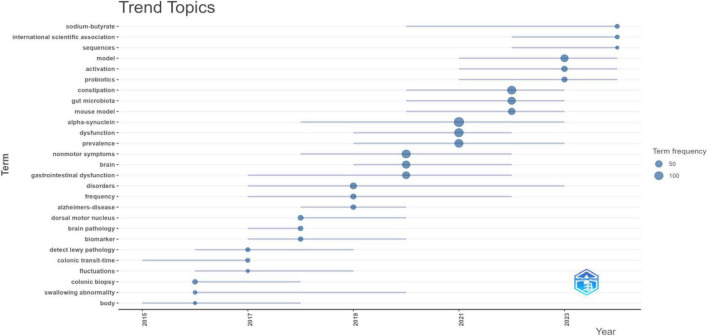
Temporal trends in keywords for PD-related GI dysfunction. The node size represents the frequency of occurrence for the corresponding topic; a higher frequency indicates a greater number of papers. Blue lines connect the evolution of the same topic across different time intervals, illustrating its developmental trajectory.

Furthermore, analysis of the topic trend map (Figure 9) reveals a shifting focus in research hotspots between 2020 and 2025. During this period, “constipation” solidified its position as a core clinical manifestation of PD-related GI dysfunction. Mechanistic investigations are increasingly centered on the roles of “gut microbiota,” “alpha-synuclein,” and metabolites such as “| sodium butyrate.” Concurrently, novel therapeutic strategies have emerged, with “probiotics” prominently featured for the therapeutic modulation of gut microbiome balance.

## 4 Discussion

### 4.1 General information related to publications

As the first bibliometric analysis focusing on gastrointestinal dysfunction (GI) in Parkinson’s disease (PD), this study systematically delineates the landscape of publication output, journal distribution, country/regional contributions, institutional collaborations, key literature, and research hotspot themes within this field from 2015 to 2025, via the construction of scientific knowledge maps. The results indicate a steady annual growth in publication volume, peaking in 2021, likely attributable to the widespread application of high-throughput sequencing and metagenomic technologies, which in turn catalyzed a surge in investigations into gut microbiota research (Qin et al., 2010). Publication output experienced slight fluctuations after 2022, a trend potentially linked to multiple factors. First, global public health crises, such as the COVID-19 pandemic, profoundly impacted all stages of medical research, spanning from basic experiments to clinical studies (Rousseau et al., 2025). Beyond these external factors, the field may also be undergoing a period of consolidation and deeper exploration, which could also contribute to extended publication cycles. Nevertheless, the continuous rise in annual citation counts reflects the field’s persistently expanding academic influence.

Further analysis reveals that countries with high scientific output are generally characterized by a high Human Development Index (HDI), with China and the United States being particularly prominent. Together, they account for 45.7% of global publications, highlighting an imbalance in the distribution of scientific resources. This high productivity can be attributed not only to well-established disease surveillance systems and abundant clinical resources but also aligns with the epidemiological trend of increasing PD prevalence correlating with higher HDI (Zhu et al., 2024). However, European nations dominate the top rankings in terms of citations per paper (see Supplementary Appendix 1), underscoring their leading role in research quality. Finland, ranking first in citations per paper, derives substantial academic impact from the University of Helsinki’s sustained research focus—initiated in 2015—on exploring the “Parkinson’s Disease-gut microbiota-clinical phenotype” research paradigm. The university has spearheaded a series of foundational studies that provide robust theoretical support for the field (Scheperjans et al., 2015).

Institutional analysis reveals that 13 out of the top 15 institutions by publication volume are located in developed countries—an advantage attributed to robust research investment and sophisticated disease screening systems, both of which facilitate the implementation of large-scale clinical studies. While Capital Medical University leads in total publication count, its relatively lower average citation rate suggests potential limitations in research quality and innovation, possibly influenced by language barriers and a lack of international collaborative networks (Table 3). Encouragingly, the collaboration network analysis (Figure 4) reveals a cohort of emerging active institutions, represented by yellow nodes—five of which are from China. This trend signifies that the field remains in a phase of rapid development, marked by strengthened international/regional cooperation. Such collaborations foster resource sharing and information exchange, enabling active participation from developing countries in this research domain.

### 4.2 Research foundation

Analysis of highly cited references (Figure 6A) indicates that the “ascending anatomical propagation” hypothesis, proposed by Braak et al. (2003a), established a key theoretical framework for understanding the pathogenesis of Parkinson’s disease (PD) originating in the peripheral nervous system. Per this hypothesis, PD-related lesions initially accumulate in specific regulatory nuclei of the lower brainstem, while sparing the substantia nigra pars compacta. This pattern offers a plausible explanation for gastrointestinal (GI) symptoms as prodromal indicators of PD. Subsequently, Scheperjans et al. (2015) provided the first demonstration in PD patients of an association between the gut microbiome and motor phenotypes. Although the temporal sequence of this association and causal mechanisms remained unclear, their work furnished early human-derived evidence supporting the concept of a “microbiota–gut–brain axis.” Sampson et al. (2016) utilized a PD animal model to demonstrate that gut microbiota is essential for the aggregation and spread of α-syn pathology, microglial activation, and development of motor deficits. They further proposed, for the first time, that “gene–microbiome interactions” could constitute a potential etiology of PD, thereby laying a theoretical foundation for the development of microbiota-targeted immunomodulatory strategies. These studies not only furnished critical microbial evidence in support of Braak’s hypothesis but have also been cited more frequently than the original work, underscoring the central role of gut microbiota in contemporary PD research. Additionally, in a systematic review published in *Lancet Neurology*, Fasano et al. (2015) summarized the clinical manifestations of GI dysfunction throughout the entire digestive tract in PD patients. They posited that GI symptoms are likely to play a significant role in PD pathophysiological subtyping, early diagnosis, and the development of targeted therapeutic approaches. It is essential to note, however, that much of this foundational evidence is derived from animal models or small-scale clinical studies, and generalizing these findings to the broader PD population remains controversial. Future large-scale clinical investigations will be crucial in bridging the gap between animal models and clinical translation.

### 4.3 Research hotspots and developments

Keyword clustering and thematic trend analysis have facilitated the identification of research hotspots and the tracking of the evolving frontiers over the past decade. In the study of GI dysfunction in PD, research has converged into three major thematic clusters centered on “mechanisms–phenotypes–interventions.”

#### 4.3.1 Mechanistic insights

At the mechanistic level, research on the gut-origin hypothesis of α-syn pathology has evolved from theoretical speculation to experimental validation. Studies by Kim et al. (2019) and Challis et al. (2020) provided the first comprehensive evidence in mouse models that pathological α-syn can propagate from the gut to the brain via the vagus nerve, with transmission efficiency showing age-dependent effects. Notably, only older mice developed neuropathological and behavioral alterations that closely recapitulate the staging observed in human postmortem studies—a finding that has since shifted focus toward aging-related lysosomal dysfunction. Nevertheless, findings from an autopsy study showed that the abundance of α-syn is actually reduced in the GIT of end-stage PD patients (Harapan et al., 2020). This key observation highlights the importance of considering the temporal dynamics of α-syn in relation to neuronal loss during PD progression, challenging its simplistic classification as a static pathological hallmark (Goedert et al., 2017). The field has thus moved beyond merely confirming the feasibility of gut-initiated pathology spread, shifting focus toward detailed molecular mechanisms and synergistic contributing factors.

In recent years, the gut microbiome and its metabolites have emerged as central regulators of gut–brain communication, with roles extending beyond a simplistic “beneficial versus harmful” dichotomy (Tan et al., 2022; Ngana et al., 2025). Multiple studies have identified characteristic gut dysbiosis in PD patients, including decreased abundance of butyrate-producing bacteria, compromised gut barrier integrity, and subsequent systemic inflammation—differences that can persist for at least 2 years after initial assessment (Li et al., 2017; Aho et al., 2019). Bhattarai et al. (2021) proposed that gut dysbiosis may not serve as an essential trigger of PD pathology but rather a catalyst accelerating disease progression. This perspective requires a refined understanding of the gut microbiome’s role in PD, necessitating further investigation into how specific bacterial strains, under particular microenvironmental conditions, may drive either pathogenic or neuroprotective responses (Tan et al., 2022; Park et al., 2025). Furthermore, *Helicobacter pylori* (*H. pylori*) infection in the upper GIT has been implicated in the pathogenesis of PD and may interfere with levodopa bioavailability (Nyholm and Hellström, 2021). Nevertheless, whether *H. pylori* exerts a protective or detrimental effect in PD remains a topic of ongoing debate (Zhao et al., 2024; Xiao et al., 2025). Although much of the research on PD-related GI dysfunction revolves around the “microbiota–gut–brain axis,” dysbiosis alone cannot fully account for the spectrum of gastrointestinal symptoms in PD. A comprehensive understanding will therefore require integration of factors such as α-syn pathology, impaired central regulation, and other complex mechanisms.

#### 4.3.2 Clinical phenotypes

Dysphagia and constipation are among the most clinically significant GI manifestations of PD, with implications extending far beyond their primary symptoms (Arora et al., 2025; Umamahesan et al., 2025). Dysphagia, in particular, is highly prevalent and closely linked to serious complications such as aspiration, pneumonia, and elevated mortality risk (Cosentino et al., 2022). Often underreported by patients, dysphagia can emerge even in the early disease stages and frequently persists throughout the disease course (Pflug et al., 2018). A large meta-analysis encompassing over 20,000 individuals reported a pooled prevalence as high as 36.9% (Gong et al., 2022). These findings underscore that clinical evaluation should not depend solely on subjective reporting, and early instrumental assessment—such as fiberoptic endoscopic evaluation of swallowing (FEES)—is strongly recommended (Labeit et al., 2024; Şirin Ahısha et al., 2025).

Constipation is the most frequent non-motor symptom in the prodromal phase of PD and increases in prevalence as the disease progresses (Yu et al., 2018; Bharucha et al., 2025). Notably, Grillo et al. (2022) observed that PD patients with constipation tend to display more severe motor symptoms and greater blood-brain barrier impairment, suggesting that constipation may signal a more aggressive disease subtype. The pathophysiology of constipation in PD is multifactorial, involving neurohumoral dysregulation, alterations in gut microbiota, and medication-related effects, among other contributing factors (Xu et al., 2022). Multimodal physiological studies indicate that approximately 60% of constipated PD patients exhibit both delayed colonic transit and anorectal dysfunction, which can be further categorized into subtypes such as impaired colonic motility, rectal hyposensitivity, and dyssynergic defecation (De Pablo-Fernández et al., 2019). These findings underscore the need for individualized assessment strategies tailored to distinct phenotypic profiles to guide targeted therapeutic interventions.

It is important to acknowledge that while the clustering results of this study prominently highlight dysphagia and constipation, the clinical spectrum of GI dysfunction in PD extends far beyond these conditions. For instance, drooling, gastroparesis, and delayed gastric emptying are equally common upper GI symptoms. These symptoms not only severely impact patients’ daily quality of life but also directly contribute to motor fluctuations by affecting drug absorption (Sharma et al., 2025). Furthermore, the gut microbiota and its metabolites, along with polymorphisms in the host COMT genotype, modulate the metabolism and therapeutic stability of levodopa-based antiparkinsonian medications (Chaparro-Solano et al., 2025; Zhang et al., 2025), representing a key driver for advancing precision medicine. Future research should build upon the clarification of core PD phenotypes to explore the complex interplay between pathophysiology and pharmacology, ultimately facilitating the construction of a more comprehensive pathological map of the PD-associated microbiome-gut-brain axis.

#### 4.3.3 Interventions and translation

The translation of microbiome research into clinical interventions for PD has gained considerable momentum. Strategies such as fecal microbiota transplantation (FMT) and probiotic supplementation are emerging as promising therapeutic avenues (Bruggeman et al., 2024). FMT entails the transfer of gut microbiota from healthy donors to patients with PD, aiming to rapidly restore a balanced intestinal ecosystem. Preclinical studies suggest that such restoration may confer neuroprotective benefits, including reinforcement of the blood-brain barrier, suppression of neuroinflammation in the substantia nigra, and protection of dopaminergic neurons via the gut-brain axis (Zhao et al., 2021; Wang et al., 2025). These benefits may alleviate both gastrointestinal and motor symptoms. Nevertheless, the clinical application of FMT in PD remains in its infancy with reported clinical outcomes showing considerable variability (Nabil et al., 2025). Consequently, future large-scale, rigorous trials are essential to definitively establish its efficacy, optimize delivery protocols, and identify patient subgroups most likely to benefit.

Probiotic interventions represent another actively explored approach. Meta-analyses indicate that various probiotic formulations can consistently ameliorate gut dysbiosis, reduce systemic inflammation, and improve PD-related GI symptoms (Tan et al., 2021), despite variations in bacterial composition across studies. The therapeutic potential of probiotics may also extend beyond monotherapy. A study by Zhou et al. (2023) showed a synergistic effect when probiotic supplementation was combined with human mesenchymal stem cells (hMSCs) in a PD mouse model. This suggests that probiotics could act as adjunctive agents to enhance other regenerative therapies through multi-target mechanisms.

Beyond these microbiome-targeted strategies, non-pharmacological interventions are also being explored, including central neuromodulation, dietary modifications, and traditional practices such as acupuncture (Smith-Hublou et al., 2024; Prasad et al., 2025). In recent years, acupuncture has demonstrated potential as a non-pharmacological intervention for improving PD-related GI dysfunction, likely via multi-target regulation of the “microbiome-gut-brain axis” (Zang et al., 2025). Animal studies reveal that electroacupuncture not only corrects gut microbiota imbalance but also activates central cholinergic pathways, promotes the release of neurotrophic factors by enteric glial cells, and exerts dopaminergic neuron protective functions (Zhang et al., 2024; Quan et al., 2025). Despite these encouraging findings, the precise molecular mechanisms remain unclear. Future well-designed clinical trials are needed to validate its efficacy and optimize treatment parameters.

### 4.4 Limitations

This study has several limitations. First, the bibliometric analysis relied exclusively on WoSCC, which may have omitted relevant literature from other databases. However, given WoSCC’s broad coverage of high-impact journals, this limitation is unlikely to substantially affect the main findings. To enhance the reliability of identified research trends, PubMed was employed as an external validation dataset. Second, due to frequent issues with author name disambiguation, author-based analyses were not performed to avoid misattribution.

### 4.5 Challenges and outlook

Although the “microbiome-gut-brain axis” has opened up a new perspective for understanding PD-related GI dysfunction, this field continues to face substantial challenges in advancing from basic research to clinical translation. At the mechanistic exploration level, current research is constrained by the gap between correlation and causation, as well as the limitations of animal models in replicating human PD pathology. Although mouse models have demonstrated the feasibility of pathological α-syn spreading from the gut to the brain via the vagus nerve, these models cannot fully reproduce the complex pathological processes of human PD, particularly sporadic forms arising from the interplay of genetic and multiple environmental factors. Furthermore, the paradoxical finding of reduced α-syn levels in the GIT of late-stage PD patients underscores the urgent need to develop novel animal models that better simulate the dynamic progression of human PD. Concurrently, advancing technologies such as single-cell sequencing are poised to play a pivotal role in elucidating causal relationships between α-syn and neuronal survival, representing a key focus for future mechanistic research.

Regarding clinical translation, while microbial intervention strategies—such as FMT and probiotics—show immense therapeutic potential, their practical clinical application is hindered by significant barriers. First, the same donor microbiota yields vastly different therapeutic outcomes across individuals, making it imperative to identify reliable biomarkers for precise selection of potential responders and thereby improving clinical efficacy. Second, the lack of standardized clinical protocols represents another major bottleneck. For instance, in FMT, key parameters such as donor microbiota selection, preparation equipment, transplantation methods, and dosage remain unstandardized. Similarly, the precise mechanisms underlying probiotic therapy remain unclear, and long-term safety and potential risks lack robust evidence from large-scale datasets. Future clinical research must transition from a “one-size-fits-all” paradigm to personalized precision treatment. Only by overcoming these challenges can the “microbiome-gut-brain axis” theory be transformed into an effective strategy for improving the quality of life for PD patients.

## 5 Conclusion

This study presents the first bibliometric analysis of publications from 2015 to 2025 focused on gastrointestinal tract (GIT) dysfunction in Parkinson’s disease (PD), systematically mapping publication trends, geographic contributions, and emerging research themes in this domain. Over the past decade, the field has evolved rapidly, shifting from initial symptom characterization to a more integrated exploration of molecular mechanisms, clinical manifestations, and translational opportunities. Future research should prioritize well-designed, large-scale clinical trials that rigorously assess the efficacy and safety of novel interventions targeting gut microbiota, α-syn dynamics, and related pathways. By synthesizing a decade of research, this study not only delineates the current intellectual landscape of PD-associated GIT dysfunction but also provides a foundational framework to guide future precision-based research and therapeutic development.
