# Supplementary material for: Development and emerging trends in gastrointestinal dysfunction of Parkinson’s disease: a decade-long bibliometric analysis
Source: Front Aging Neurosci. 2025 Nov 28;17:1712302. doi: 10.3389/fnagi.2025.1712302 (PMC12698610; doi:10.3389/fnagi.2025.1712302)
Supplement: Supplementary file 2 [file Table_2.docx]

| **Co-cited journals** | **Citations** | **IF(2024)** |
| --- | --- | --- |
| MOVEMENT DISORDERS | 4804 | 7.6 |
| PARKINSONISM AND RELATED DISORDERS | 2515 | 3.4 |
| NEUROLOGY | 1612 | 8.5 |
| PLOS ONE | 1104 | 2.6 |
| ACTA NEUROPATHOLOGICA | 991 | 9.3 |
| JOURNAL OF PARKINSON'S DISEASE | 824 | 5 |
| NEUROBIOLOGY OF DISEASE | 739 | 5.6 |
| ANNALS OF NEUROLOGY | 648 | 7.7 |
| NEUROGASTROENTEROLOGY AND MOTILITY | 760 | 2.9 |
| LANCET NEUROLOGY | 728 | 45.5 |

**Top 10 Journals with the Highest Number of Co-Citations**
